# Public health round-up

**DOI:** 10.2471/BLT.26.010826

**Published:** 2026-08-01

**Air pollution and health**

The World Health Organization has launched updated data on the sustainable development goal (SDG) indicators tracking the link between air pollution and health. New data reveal persistent inequalities in air pollution exposure and health impacts: while fine particulate matter (PM2.5) levels dropped globally until 2020, they have since remained largely unchanged, with low- and middle-income countries facing significantly higher exposure risks than high-income nations. Exposure to both ambient and household air pollution affects the severity of noncommunicable diseases, including heart disease, stroke, chronic obstructive pulmonary disease and lung cancer, and impacts the most vulnerable communities and at-risk populations.

**Figure:**
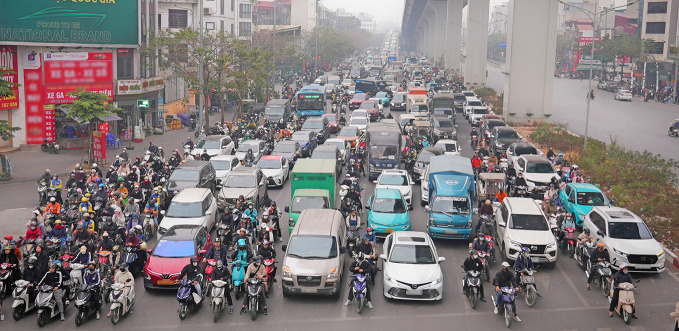
WHO/Hồ Chí Cường

## Guidelines on cognitive decline and dementia

The World Health Organization (WHO) has released updated guidelines on reducing the risk of cognitive decline and dementia, offering countries evidence-based recommendations to help prevent or delay the condition throughout the life course. Dementia currently affects more than 57 million people worldwide, with nearly 10 million new cases diagnosed each year. Alzheimer disease accounts for an estimated 60% of all cases.

"We know more today than ever before about what drives dementia risk, and these guidelines translate that knowledge into action," said Tedros Adhanom Ghebreyesus, WHO Director-General. "Countries now have clear, evidence-based recommendations they can put into practice immediately to protect people's cognitive health."

Although there is no cure for dementia, WHO notes that up to 45% of cases may be linked to modifiable risk factors, including tobacco and alcohol use, physical inactivity, social isolation, air pollution, high blood pressure and diabetes. The updated guidelines consolidate the latest evidence on healthy behaviours, medical interventions and environmental measures that can reduce dementia risk.

Recommendations include increasing physical activity, adopting a healthy diet, stopping tobacco use, reducing harmful alcohol consumption, managing hypertension, diabetes and high cholesterol, and reducing exposure to air pollution. WHO also highlights cognitive training, social engagement and the use of hearing aids as potential risk-reduction measures. 

https://bit.ly/4aXRbkE

## Clinical trial for Bundibugyo virus disease

Patient enrolment begun in July in the PARTNERS (Platform Adaptive Randomised Trial for New and Repurposed Filovirus TreatmentS) clinical trial, a major international effort to evaluate potential treatments for Bundibugyo virus disease in the Democratic Republic of the Congo.

Sponsored by WHO and coordinated by partners including the *Institut National de Recherche Biomédicale* (INRB), Democratic Republic of the Congo, the Institute of Tropical Medicine, Belgium and the University of Oxford, United Kingdom of Great Britain and Northern Ireland, the study will assess whether the antiviral therapies MBP134 and remdesivir can improve survival among people diagnosed with Bundibugyo virus disease, including whether a combination of both treatments offers additional benefits.

Since May 2026, when WHO declared the outbreak a Public Health Emergency of International Concern, more than 1400 laboratory-confirmed cases and 438 deaths have been reported. While effective treatments exist for some forms of Ebola virus disease, no therapies are currently approved for Bundibugyo virus disease.

The trial will enrol patients of all ages with confirmed infection and provide supportive clinical care alongside investigational treatments. Designed as a platform trial, PARTNERS can incorporate additional therapies as they become available. Findings will be monitored by an independent data and safety board as the trial progresses.

https://bit.ly/4yqJbTn

## First diagnostic test for Bundibugyo virus disease

The first molecular diagnostic test for Bundibugyo virus disease has been added to WHO’s Emergency Use Listing (EUL), marking an important step in strengthening outbreak detection and response. The test identifies the virus’s genetic material in blood samples, enabling rapid and accurate confirmation of infection. The listing comes as countries respond to the largest recorded outbreak of Ebola disease caused by Bundibugyo virus. 

"Public health emergencies require not only speed, but also confidence that the health products being used meet standards for quality, safety and performance," said Yukiko Nakatani, WHO Assistant Director-General for Health systems, access and data. "During a fast-moving outbreak, timely access to quality-assured diagnostic tests can make a critical difference in containing transmission. Through this Emergency Use Listing, WHO is helping countries access trusted diagnostic tools more rapidly so that they can respond more effectively.”

WHO’s EUL mechanism helps accelerate access to essential health products by assessing their quality, safety and performance during public health emergencies. Laboratory testing capacity in affected areas has expanded significantly, from a few sites capable of conducting up to 400 tests daily to a network of 10 laboratories with a combined capacity exceeding 2000 tests per day.

With support from WHO and the Africa Centres for Disease Control and Prevention (Africa CDC), laboratory testing capacity has expanded from a limited number of sites, primarily *Institut National de Recherche Biomédicale* in Kinshasa and Goma, with an estimated combined capacity of approximately 200–400 tests per day, to a broader network of 10 laboratories across affected provinces, with a reported capacity of over 2000 tests per day. 

https://bit.ly/4fH2HU8

## Global childhood immunization 

Global childhood immunization coverage continued its slow recovery in 2025, according to new WHO and UNICEF estimates, with 90% of infants receiving at least one dose of the diphtheria, tetanus and pertussis (DTP) vaccine and 85% completing the full three-dose series. While both figures increased by one percentage point from the previous year, coverage remains below pre-pandemic levels and progress has largely stagnated over the past decade.

The number of “zero-dose” children, those who did not receive a single vaccine during their first year of life, fell by nearly 750000 to 13.5 million. However, concerns remain over the growing number of children who begin vaccination schedules but fail to complete them. An estimated 7.3 million infants received their first DTP dose but did not receive their first measles vaccine, contributing to measles coverage rates that remain well below the 95% threshold needed to prevent outbreaks. As a result, 57 countries reported measles outbreaks in 2025.

Conflict, displacement, poverty and vaccine hesitancy continue to undermine progress. More than half of all zero-dose children live in fragile and conflict-affected settings, while some middle- and high-income countries have also experienced declining coverage.

WHO and UNICEF warn that funding pressures, weakening health data systems and misinformation could threaten future gains, and are calling for renewed investment in immunization programmes, disease surveillance and efforts to reach children in the most vulnerable communities.

https://bit.ly/3QWo5LS

## Global report on cancer 

WHO and the International Agency for Research on Cancer (IARC) have released the *Global status report on cancer 2026*, warning that annual cancer cases could rise from 20.6 million today to nearly 35 million by 2050 without urgent action. Cancer remains the world’s second leading cause of death, claiming close to 10 million lives each year.

The report highlights stark inequalities in cancer outcomes. While 87% of women diagnosed with breast cancer survive at least five years in high-income countries, survival drops to around 42% in low-income countries. Fewer than one third of countries currently include cancer care in their universal health coverage packages, limiting access to prevention, diagnosis, treatment and supportive care for millions.

Lung cancer remains the leading cause of cancer death worldwide, while nearly four in ten cancer cases globally are linked to preventable risk factors, particularly infections such as human papillomavirus (HPV), hepatitis B and C, alcohol and tobacco use, high body mass index and insufficient physical activity.

"While we are seeing reductions in some cancer rates in countries that have implemented prevention policies, progress has been too slow," said Elisabete Weiderpass, director of IARC. "The cancer profile is evolving, increasingly driven by rising rates of obesity, physical inactivity, unhealthy diets and air pollution. Cancer prevention must remain a political priority."

https://bit.ly/3RntQ5l

## Climate and migration 

WHO’s Western Pacific Regional Office has launched a new regional research agenda on health, migration and displacement in the region, with a particular focus on the impacts of climate change. 

Key research priorities include advancing universal health coverage and primary health care, improving the inclusion of migrants and displaced populations in emergency preparedness and response, and examining the social, economic, environmental and structural factors that shape health outcomes.

“Climate change, migration, displacement and health can no longer be considered separate issues. Across the Western Pacific Region, their impacts are increasingly visible in communities and health systems. This agenda provides an important foundation for generating the evidence needed to inform equitable and effective responses,” said Sandro Demaio, director and head of office of the WHO Asia-Pacific Centre for Environment and Health.

https://bit.ly/4poiTgi

**Cover photo**

Teams from the Venezuelan Ministry of Health and the Pan American Health Organization conduct a field visit to assess infrastructure conditions following the June 24 earthquake in La Guaira, Venezuela.

**Figure:**
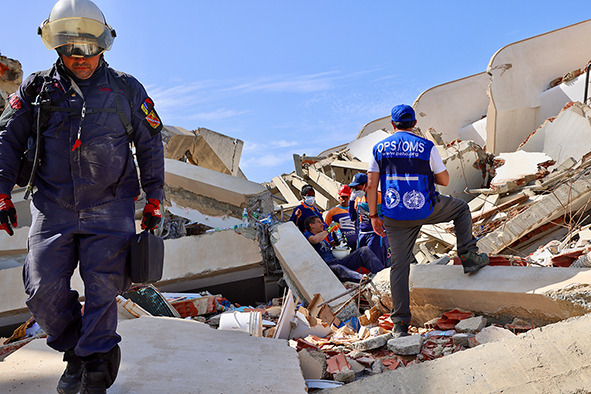
AMRO/PAHO

**Looking ahead**

8–22 September. 81^st^ session of the United Nations General Assembly. New York, United States of America. https://bit.ly/4w4uvaV

17 September. World Patient Safety Day. https://bit.ly/4vBHZd9

